# Using three scenarios to explain life expectancy in advanced cancer: attitudes of patients, family members, and other healthcare professionals

**DOI:** 10.1007/s00520-022-07167-3

**Published:** 2022-06-15

**Authors:** Sharon H. Nahm, Martin R. Stockler, Andrew J. Martin, Peter Grimison, Peter Fox, Rob Zielinski, Geoffrey AT. Hawson, Martin HN. Tattersall, Belinda E. Kiely

**Affiliations:** 1 The NHMRC Clinical Trials Centre, The University of Sydney, Locked Bag 77, Camperdown, Sydney, NSW 1450 Australia; 2 Sydney Medical School, The University of Sydney, Sydney, Australia; 3 Concord Cancer Centre, Sydney, Australia; 4 Chris O’Brien Lifehouse, Sydney, Australia; 5 Alan Coates Cancer Centre, Dubbo, Australia; 6 Central West Cancer Care Centre, Orange, Australia; 7 Nambour General Hospital, Nambour, Australia; 8 Macarthur Cancer Therapy Centre, Sydney, Australia

**Keywords:** Prognosis, Prognostic discussions, Life expectancy, Doctor-patient communication, Scenarios for survival, Advanced cancer

## Abstract

**Aim:**

To evaluate a web-based tool for estimating and explaining three scenarios for expected survival time to people with advanced cancer (patients), their family members (FMs), and other healthcare professionals (HCPs).

**Methods:**

Thirty-three oncologists estimated the “median survival of a group of similar patients” for patients seeking quantitative prognostic information. The web-based tool generated worst-case, most likely, and best-case scenarios for survival based on the oncologist’s estimate. Oncologists presented the scenarios to each patient and provided a printed summary to patients, FMs, and HCPs. Attitudes to the information were assessed by questionnaires. Observed survival for each patient was compared with the oncologist’s estimated survival and the three scenarios.

**Results:**

Prognosis was discussed with 222 patients: median age 67 years; 61% male; most common primary sites pancreas 15%, non-small-cell lung 15%, and colorectal 12%. The median (range) for observed survival times was 9 months (0.5–43) and for oncologist’s estimated survival times was 12 months (2–96). Ninety-one percent of patients, 91% of FMs, and 84% of HCPs agreed that it was helpful having life expectancy explained as three scenarios. The majority (77%) of patients judged the information presented about their life expectancy to be the same or better than they had expected before the consultation. The survival estimates met a priori criteria for calibration, precision, and accuracy.

**Conclusions:**

Patients, FMs, and HCPs found it helpful to receive personalized prognostic information formatted as three scenarios for survival. It was feasible, acceptable, and safe to use a web-based resource to do this.

**Supplementary Information:**

The online version contains supplementary material available at 10.1007/s00520-022-07167-3.

## Introduction

Conversations about prognosis are important for people affected by incurable cancer and help patients and their families make decisions about treatment, plans for the future, and choices about end-of-life care. Patients who have a good understanding of their life expectancy are less likely to choose futile treatments and aggressive medical interventions toward the end of life; are more likely to accept palliative care services earlier; have better quality of life at the end of life; and have surviving carers with better quality of life during the bereavement period [1–3].

Despite this, many oncologists provide patients with minimal information about life expectancy or avoid such discussions altogether [4–6]. Reported reasons for this include fear of upsetting patients, fear of providing inaccurate information, and insufficient training in prognostication [7, 8]. As a consequence, many patients do not fully understand their situation and goals of treatment. Conversations about prognosis are often deferred until the last months of life, robbing patients and families the opportunity to plan and discuss their wishes while they are well. When conversations about prognosis do occur, they are rarely documented in the patient’s medical record or letters to other healthcare professionals [9, 10]. This makes it difficult for all healthcare professionals involved in the patient’s care to know what has been discussed and may result in patients receiving inconsistent information.

Most patients want some information about their expected survival time, and many want information about specific scenarios, for example, the longest survival with treatment, average survival, and shortest survival without treatment [11–13]. We previously surveyed 505 people with a cancer experience about their preferred format for presenting information on expected survival time to a hypothetical patient with advanced cancer and found that 88% preferred three scenarios for survival (worst-case, most likely, and best-case), and only 5% preferred a single estimate of the median survival time [14].

In previous work, we showed that certain percentiles of an overall survival (OS) curve provide a useful basis for estimating three scenarios for survival [15]. For example, the 90th percentile, the time when 90% of people are still alive and 10% have died, can approximate the worst-case scenario (shortest 10% of survival times) and the 10th percentile, the time when 10% of people are still alive and 90% have died, can approximate the best-case scenario (longest 10% of survival times). We have also shown that simple multiples (0.25, 0.5, 2, and 3) of an OS curve’s median can be used to estimate its percentiles [15–23]. To illustrate, the 90th percentile (representing the upper bound of the worst-case scenario) is approximately one-quarter of the median OS; the 75th percentile (lower bound of the most likely scenario) is approximately half the median OS; the 25th percentile (upper bound of the most likely scenario) is approximately double the median OS; and the 10 percentile (lower bound of the best-case scenario) is approximately three times the median OS. For example, if the median OS is 12 months then the worst-case scenario is less than 3 months (0.25 x 12), the most likely scenario is 6 to 24 months (0.5 to 2 x 12), and the best-case scenario is 36 months or longer (3 x 12).

Using these simple rules of thumb, we developed a web-based tool (iTool) to help oncologists estimate and explain individualized scenarios for survival to patients with incurable cancer seeking quantitative information about their prognosis. With consumer input, we developed a one-page summary to help explain this information to patients, family members or carers (FMs), and other healthcare professionals (HCPs).

The aim of this study was to evaluate the iTool for estimating and explaining personalized information about life expectancy in people with advanced cancer, their medical oncologists, FMs, and other HCPs.

## Patients and methods

We conducted a multi-site, single-arm, phase 2 trial. The target population was adults with incurable cancer attending the clinic of a participating medical oncologist and indicating that they wanted quantitative information about their prognosis (either spontaneously or when their oncologist offered to discuss prognosis). FMs were eligible if present during the consultation when life expectancy was discussed. HCPs involved in a participating patient’s care and receiving letters from the oncologist as standard of care (e.g., general practitioner, referring surgeon, radiation oncologist) were also invited to participate.

Participating oncologists were provided with access to the iTool for the duration of the study (available at https://ctc.usyd.edu.au/3scenarios/*)*. When a patient wanted information on their expected survival time, we asked the oncologist to estimate the patient’s life expectancy defined as “the median survival of a group of similar patients” based on studies of patients in the same situation, prognostic tools, or their personal clinical experience. The iTool calculated ranges for the three scenarios using simple multiples of the oncologist’s estimate based on our previous work [16]. This information was printed for the patient and FMs to take home (Supplementary Text 1), and copies were placed in the patient’s medical record and sent to HCPs with the oncologist’s standard letter.

Following the consultation, the oncologist entered the patient demographics and cancer details and completed a questionnaire about the perceived usefulness of the iTool for that patient, including if it was helpful, easy to use, stressful, or lengthened the consultation (Supplementary Text 2). After providing written, informed consent, patients completed a questionnaire about the prognostic information they received (Supplementary Text 3). FMs and HCPs completed similar questionnaires regarding the information they received (Supplementary Text 4 and 5). The study was approved by the health research ethics committee at all participating sites.

The primary measure of effect was the proportion of patients who agreed or strongly agreed that “having my life expectancy explained this way was helpful.” Other measures of effect included the proportions of patients who agreed or strongly agreed that “Having my life expectancy explained this way”: made sense, gave hope, or made them feel worried or anxious. Patients were asked if the information about their life expectancy was better, worse, or about the same as they had thought before the consultation. Patients were also asked if they agreed it was helpful to be told each of the scenarios (best-case, worst-case, and most likely).

Patients also completed three other questionnaires:

1. The Short State Trait Anxiety Inventory (STAI), a six-item short form of the state scale of the Spielberger STAI [24]
2. The Herth Hope Index, a 12-item adapted version of the Herth Hope Scale with three subscales measuring temporality and future, positive readiness and expectancy, and interconnectedness [25]
3. The Life Orientation Test Revised (LOTR), a 10-item scale measuring levels of optimism [26]

A higher score indicated greater levels of either anxiety, hope, or optimism with each scale, respectively.

At the end of the study, participating oncologists completed a second questionnaire to determine their attitudes to using the iTool when discussing prognosis with their patients (Supplementary Text 6). Oncologists were sent emails asking them to update the survival status of each patient at time points corresponding to simple multiples of their estimated median survival time: 0.25, 0.5, 1.0, 2.0, 3.3, and 5.

For comparability with previous studies, we defined a point estimate of life expectancy (estimated median survival time of a group of similar patients) as precise if it was within 0.67 to 1.33 times the observed survival time [15] and hypothesized that approximately 20% to 30% of estimates would meet this definition. For each patient, we calculated the ratio of the observed survival time (OST) to their oncologist’s estimated survival time (EST) and used the Kaplan–Meier distribution of the ratio (OST/EST) to account for censored observations (patients still alive at their last follow-up). We expected oncologists’ EST to be well-calibrated (i.e., approximately equal proportions (50%) being longer than the observed survival time (OST/EST < 1) and shorter than the observed survival time (OST/EST > 1)).

Based on the broad concept of accuracy used in our previous work, we also hypothesized that approximately:

Five to ten percent of patients would die within one-quarter of their oncologists’ estimate (OST/EST ≤ 0.25).

Fifty percent of patients would have a survival time within half to double their oncologists’ estimate (0.5 ≤ OST/EST ≤ 2).

Ten percent of patients would live beyond three times their oncologist’s estimate (OST/EST ≥ 3) [20].

A sample of size of at least 70 patients was calculated to provide > 95% power to distinguish the observed proportion of patients finding the iTool helpful from hypothetical true proportions of 80% or more versus 60% or less with an allowance of 20% for incomplete data. Associations between baseline characteristics and responses to questions about the prognostic information were assessed with logistic regression. Statistical analyses were done using R version 4.0.4.

## Results

**Fig. 1:**
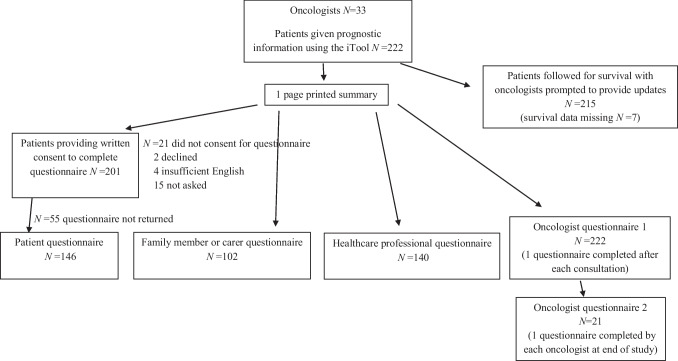
Consort diagram

Between August 2012 and May 2016, 33 oncologists used the iTool to explain life expectancy to 222 patients (Fig. 1), of whom 201 consented to participate in the evaluation part of the study and 146 returned completed questionnaires (response rate 73%). Completed questionnaires were returned by 102 FMs and 140 HCPs. All 33 participating oncologists completed a questionnaire following each patient consultation (oncologist questionnaire 1), and 21 of the 33 oncologists completed a questionnaire at the end of the study (oncologist questionnaire 2).

The baseline characteristics of participating patients are summarized in Table 1. The median age was 67 years, and the majority (61%) were male. For most patients, the iTool was used more than 8 weeks after their diagnosis of incurable cancer, and at a third or subsequent consultation. The median estimated survival time was 12 months (range 2 to 96 months).

**Table 1:** Patient baseline characteristics (*N* = 222)

Characteristic	*N* (%)^a^
Median age, years (range)	67 (27–90)
Sex, male	137 (61)
Education *(available for 146 patients who completed the patient questionnaire)*	
Year 10 or less	
High school/post high school qualification Unknown	58 (40) 81(55) 7 (5)
Spoken English level	
Fluent/Native Adequate Poor	211 (95) 7 (3) 4 (2)
ECOG performance status	
0 1 2 3	46 (21) 130 (59) 43 (19) 3 (1)
Cancer type	
Pancreatic Non-small cell lung cancer Colorectal Prostate Breast Kidney Other	34 (15) 34 (15) 26 (12) 25 (11) 19 (9) 17 (8) 67 (30)
Time from diagnosis of incurable cancer	
≤ 8 weeks > 8 weeks	99 (45) 123 (55)
Consultation number	
Initial Second Third or subsequent	65 (29) 26 (12) 131 (59)
Estimated survival time	
< 3 months 3– < 9 months 9–15 months > 15 months	3 (1) 81 (36) 72 (32) 66 (30)

^a^Unless otherwise specified

The vast majority (91%) of patients agreed or strongly agreed that the survival information presented by the iTool was helpful (Table 2). More patients preferred to hear each of the three scenarios (worst-case 81%, most likely 86%, and best-case 92%) than to be told the estimated median survival (78%). Seventy-seven percent of patients responded that the information about their prognosis explained as three scenarios for survival was about the same as, or better than, they expected before discussing it with their oncologist.

**Table 2:** Attitudes of patients to receiving personalized scenarios for survival using the web-based tool (*N* = 146)

Proportion who agree^a^ with each statement	*N* (%)	95% CI
Having my life expectancy explained this way:		
Is helpful	126/138 (91)	85–95
Makes sense	136/142 (96)	91–98
Helps them make plans	126/144 (88)	81–92
Gives them hope	78/140 (56)	47–64
Reassures them	89/139 (64)	56–72
Improves their understanding	122/138 (88)	82–93
Makes them feel worried or anxious	41/138 (30)	23–38
Upsets them	58/140 (41)	34–50
Would be useful for their family members	107/142 (75)	68–82
Would be useful for their family doctor	127/141 (90)	84–94
Being told the following scenario was helpful:		
Best-case	131/142 (92)	87–96
Most likely	122/142 (86)	79–91
Worst-case	115/142 (81)	74–87
Being told the time half a group of people would live longer or shorter than was helpful	110/141 (78)	70–84
Preference for scenario to be told about first:		
Best-case	28/143 (20)	14–27
Most likely	49/143 (34)	27–43
Worst-case	14/143 (10)	6–16
Order is not important to me	52/143 (36)	29–45
How life expectancy information compared to expectations		
Better than expected	44/142 (31)	24–39
About the same as expected	66/142 (46)	38–55
Worse than expected	32/142 (23)	16–30
It was helpful for me to receive a printed summary of this information	127/140 (91)	85–94

^a^Includes agree and strongly agree responses

The attitudes of oncologists, HCPs, and FMs to having the information estimated and explained this way are summarized in Table 3. The median number of times each oncologist used the iTool was four (range 1 to 50). In 96% of consultations, oncologists agreed or strongly agreed that explaining life expectancy as three scenarios was helpful. There were very few consultations where oncologists agreed or strongly agreed that using the iTool significantly lengthened the consultation (9%). The majority of HCPs agreed or strongly agreed that having the information presented this way would be helpful for themselves (84%) and their patients (73%) and was more informative than the prognostic information they usually received (88%).

**Table 3:** Attitudes of family members and carers, healthcare professionals, and oncologists to the information provided by the web-based tool

	***N* (%)**	**95% CI**
**Family members and carers (*N* = 102)**		
Proportion who agree^**a**^ that the survival information presented by the iTool		
Is helpful	92/101 (91)	84–95
Is reassuring	51/100 (51)	41–61
Makes sense	97/101 (96)	90–98
Helps them make plans	71/99 (72)	62–80
Gives them hope	56/101 (55)	46–65
Improves their understanding	85/100 (85)	77–91
Is too complicated	9/99 (9)	5–16
Is upsetting	47/98 (48)	38–58
**Healthcare professionals (*N* = 140)**		
Proportion who agree^a^ that the survival information presented by the iTool		
Is helpful for themselves	115/137 (84)	77–89
Is helpful for patients	98/134 (73)	65–80
Is reassuring for patients	37/131 (28)	21–37
Is distressing for patients	51/135 (38)	30–46
Makes sense	122/135 (90)	84–94
Will help them make management and treatment decisions	98/138 (71)	63–78
Improves their understanding of the patients’ prognosis	103/136 (76)	68–82
Is more informative than prognostic information usually received	117/133 (88)	81–93
**Oncologists (*N* = 222 consultations)**^**b**^		
Proportion of consultations where the oncologist agreed^a^ that explaining life expectancy as 3 scenarios		
Is helpful	214/222 (96)	93–98
Is difficult	17/222 (8)	5–12
Is stressful	26/222 (12)	8–17
Is intrusive	42/222 (19)	14–25
Significantly lengthened the consultation	19/222 (9)	6–13
Was facilitated by using the iTool	207/222 (93)	89–96
Proportion of consultations where the oncologist agreed^a^ that having life expectancy explained this way *for the patient*		
Is helpful	196/222 (88)	83–92
Is reassuring	113/222 (51)	44–57
Is upsetting	36/222 (16)	12–22
Is too complicated	6/222 (3)	1–6
Improved their understanding	193/222 (87)	82–91

^a^Includes agree and strongly agree responses

^b^Number of consultations, 33 oncologists completed a questionnaire after each patient consultation

Associations between patients’ baseline characteristics and agreeing or strongly agreeing that presenting the life expectancy information as three scenarios was helpful are summarized in Table 4. Higher scores for hope were associated with a higher likelihood of agreeing that the information was helpful (OR 5.7, 95% CI 1.2 to 27, *p* = 0.03).

**Table 4:** Characteristics associated with patients agreeing that the information presented by the web-based tool was helpful (*N* = 146)

Variables	Agree or strongly agree	Disagree, strongly disagree, or unsure	Odds ratio (95% CI)	*P* value
Age, years				
< 50 50–70 > 70	14/16 (88) 63/70 (90) 49/52 (94)	2/16 (13) 7/70 (10) 3/52 (6)	ref 1.3 (0.24–6.9) 2.3 (0.35–15)	0.60
Sex				
Female Male	44/46 (96) 82/92 (89)	2/46 (4) 10/92 (11)	ref 0.37 (0.08–1.8)	0.22
Cancer type				
Prostate Kidney Non-small cell lung cancer Pancreatic Colorectal Breast Other	18/19 (95) 12/13 (92) 19/21 (91) 16/18 (89) 16/18 (89) 13/13 (100) 32/36 (89)	1/19 (5) 1/13 (8) 2/21 (10) 2/18 (11) 2/18 (11) 0/13 (0) 4/36 (11)	2.3 (0.23–22) 1.5 (0.15–15) 1.2 (0.20–7.1) 1.0 (0.17–6.1) 1.0 (0.17–6.1) NA ref	0.78
Education level				
Year 10 or less High school or above	49/55 (89) 71/76 (93)	6/55 (11) 5/76 (7)	ref 1.7 (0.50–6.0)	0.38
Short State Trait Anxiety Inventory^a^				
Below median (13)	60/65 (92)	5/65 (8)	ref	
Above median	64/70 (91)	6/70 (9)	0.89 (0.25–3.1)	0.85
Herth Hope Index^a^				
Below median (38) Above median	58/68 (85) 66/68 (97)	10/68 (15) 2/68 (3)	ref 5.7 (1.2–27)	**0.03**
Life Orientation Test Revised^a^				
Below median (16) Above median	46/51 (90) 77/84 (91)	5/51 (10) 7/84 (8)	ref 1.2 (0.36–4.0)	0.77
Estimated median survival				
< 9 months 9–15 months > 15 months	44/48 (92) 37/42 (88) 45/48 (94)	4/48 (8) 5/42 (12) 3/48 (6)	ref 0.67 (0.17–2.7) 1.4 (0.29–6.5)	0.64
Time since diagnosis of incurable cancer				
≤ 8 weeks > 8 weeks	47/50 (94) 79/88 (90)	3/50 (6) 9/88 (10)	ref 0.56 (0.14–2.2)	0.40

^a^Higher score indicated greater levels of either anxiety, hope or optimism

**Fig. 2:**
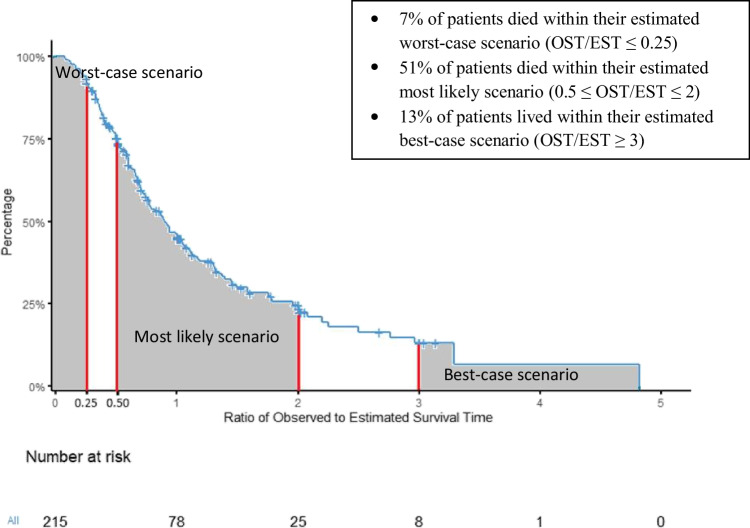
Kaplan–Meier distribution of observed-to-estimated survival time ratios (*N* = 215)

The median observed survival time was 9 months (range 0.5 to 43). Oncologists’ estimates were well-calibrated, with 54% (95% CI 46 to 61) of patients living longer than their EST and 46% (95% CI 39 to 54) living shorter than their EST. As hypothesized, 27% (95% CI 20 to 34) of oncologists’ point estimates of life expectancy met our arbitrary criterion for precision (within 0.67–1.33 times the OST). The proportions of patients with OSTs falling within prespecified ranges for the three scenarios corresponded closely with our a priori hypotheses: 7% (95% CI 3 to 10) of patients died within their estimated worst-case scenario; 51% (95% CI 43 to 59) lived within their estimated most likely scenario; and 13% (95% CI 8 to 23) lived within their estimated best-case scenario. Figure 2 shows the distribution of the OST/EST ratio for each patient.

At the end of the study, 81% (17/21) of responding oncologists agreed or strongly agreed that the iTool made discussing prognosis easier; 52% (11/21) agreed or strongly agreed that it made them more prepared to discuss prognosis; and 86% (18/21) agreed or strongly agreed that they would like to continue using the iTool beyond the study.

## Discussion

The majority of responding patients, FMs, and HCPs found it helpful to receive personalized scenarios for survival generated by the iTool. While 40% of patients found the life expectancy information upsetting, the majority of patients and FMs still found it helpful to be told each of the three scenarios, including the worst-case scenario. Most responding patients (77%) reported that the life expectancy information they received was the same or better than they had expected before the consultation. A higher score for hope was associated with higher likelihood of responding that the survival information was helpful. As hypothesized, oncologists’ point estimates of life expectancy for individual patients were well-calibrated but imprecise. However, scenarios for survival time calculated by the iTool using simple multiples of the oncologists’ estimates corresponded closely with our a priori estimates: approximately 10% of patients died within one quarter of their EST, approximately 50% lived within half to double their EST, and approximately 10% lived longer than three times their EST [16].

These favorable attitudes to using the iTool were consistent with our previous findings that people with cancer prefer to receive prognostic information formatted as three scenarios (worst-case, most-likely, best-case) rather than a point estimate of the median survival time [14]. Our previous work involved a hypothetical patient so it was reassuring to find similar results when real patients were presented with a personalized estimate of their own prognosis in this format. Participating oncologists reported that the iTool was easy to use and made them better prepared to discuss prognosis.

Our data indicate that the iTool could help overcome commonly cited barriers to discussions about prognosis including: lack of tools, lack of time, not knowing what to say, and fear of upsetting patients and family members [8, 20, 21]. Oncologists reported that using the iTool prolonged less than 10% of consultations, an important finding given fear of prolonging consultations is a reported barrier to discussing prognosis [8, 27].

Most patients found it helpful to receive a printed summary of information about their prognosis. Similarly, more than 70% of HCPs agreed that the printed three scenarios for survival information they received was more informative than the prognostic information they usually receive from oncologists, and that this information would help them make management and treatment decisions with their patients. We previously reported that quantitative information about prognosis was rarely included in letters from medical oncologists to HCPs [9]. Providing the one-page printed summary to HCPs could help ensure members of the multidisciplinary care team are aware of the estimated prognosis, and of what the patient has been told. This should improve the consistency of information and perhaps even improve patient care.

Fear of upsetting the patient is a commonly reported barrier to discussing prognosis [8]. Interestingly, oncologists in this study agreed that patients would find the prognostic information upsetting in only 16% of consultations. This may be because they perceived the format of presenting three scenarios was less upsetting or perhaps that the patients they selected for the study were those less likely to be upset by the information. We found 40% of patients agreed that the prognostic information they received was upsetting, yet despite this, over 90% agreed that the information was helpful. We previously reported that providing patients with ranges for three scenarios was judged to offer more hope than providing a single point estimate of survival [14]. It is possible that providing ranges for three scenarios helps patients understand the uncertainty of survival estimates and allows them to hope for a realistic best-case scenario. Most patients found that the information presented about their life expectancy was either about the same as, or better than, they expected, even when given their worst-case scenario. Previous studies reporting that patients with advanced cancer are more likely to over-estimate their survival compared to their oncologists have generally compared a point estimate made by the patient with a point estimate made by the oncologist [2, 21, 28]. Another possible explanation is that the majority of patients in our study (59%) received their survival time scenarios at a third or subsequent consultation, and there may have been other, earlier discussions about prognosis.

This study and approach to discussing prognosis has several key strengths. We developed an easily accessible, web-based tool designed to help oncologists explain “worst-case, most likely, and best-case scenarios for survival time” to people affected by cancer. We studied the attitudes of patients receiving personalized information about their own life expectancy and also the attitudes of oncologists, FMs, and HCPs. We included patients with a wide range of ages, cancer types, and estimated life expectancies. This supports the applicability of the iTool in people with advanced cancer seeking quantitative information about their prognosis from receptive oncologists. Our data support the accuracy of scenarios for survival time based on simple multiples of their oncologist’s estimate of life expectancy.

The main limitations of this study are the biases inherent to a single-arm design. Participating oncologists may have had greater interest and expertise in discussing prognosis and may have selected patients they judged likely to welcome information presented this way. Our response rate for patient questionnaires was 73%; the 55 consenting patients who did not return a questionnaire may have had less favorable views. We did not assess patients’ understanding of the information presented about their prognosis. Our study involved only 33 oncologists. The generalizability of these findings requires further study, especially in the current era of increasing use of immunotherapy.

### Future directions

Changing the behavior and practice of doctors is difficult. Australia has mandatory workshops on communicating prognosis for advanced trainees in medical oncology. Incorporating the iTool into this training offers an opportunity to increase its use. The www.cancersurvivalrates.com website is another useful resource for oncologists and people affected by cancer who seek quantitative information about prognosis. This website provides information based on recent data collected by the US SEER program and now provides information presented as three scenarios when the estimated median survival time is less than 3 years. These resources can help start and facilitate conversations about prognosis between patients and their doctors. Further research is needed to evaluate patients’ understanding of presented information about prognosis.

## Conclusion

We have provided strong evidence supporting the recommendation that oncologists use three scenarios for survival time when thinking and talking about prognosis in advanced cancer. It was feasible, acceptable, helpful, and safe to use a web-based resource to do this.

*Our web-based tool can be accessed via the link* https://ctc.usyd.edu.au/3scenarios/

### Supplementary Information

Below is the link to the electronic supplementary material.

Supplementary file1 (DOCX 67 KB)

## Data Availability

The authors have full control of all primary data and agree to allow the journal to review their data if requested.
