# Correction to: Using three scenarios to explain life expectancy in advanced cancer: attitudes of patients, family members, and other healthcare professionals

**DOI:** 10.1007/s00520-024-08597-x

**Published:** 2024-05-27

**Authors:** Sharon H. Nahm, Martin R. Stockler, Andrew J. Martin, Peter Grimison, Peter Fox, Rob Zielinski, Geoffrey AT. Hawson, Martin HN. Tattersall, Belinda E. Kiely

**Affiliations:** 1 The NHMRC Clinical Trials Centre, The University of Sydney, Locked Bag 77, Camperdown, Sydney, NSW 1450 Australia; 2 Sydney Medical School, The University of Sydney, Sydney, Australia; 3 Concord Cancer Centre, Sydney, Australia; 4 Chris O’Brien Lifehouse, Sydney, Australia; 5 Alan Coates Cancer Centre, Dubbo, Australia; 6 Central West Cancer Care Centre, Orange, Australia; 7 Nambour General Hospital, Nambour, Australia; 8 Macarthur Cancer Therapy Centre, Sydney, Australia

**Correction to: Supportive Care in Cancer (2022) 30:7763–7772**

10.1007/s00520-022-07167-3

Instead of

"For example, if the median OS is 12 months then the worst-case 36 months or longer (3 x 12)."

It should read:

"For example, if the median OS is 12 months then the worst-case scenario is less than 3 months (0.25 x 12), the most likely scenario is 6 to 24 months (0.5 to 2 x 12), and the best-case scenario is 36 months or longer (3 x 12)."

The original article has been corrected.

