# Supplementary material for: Using three scenarios to explain life expectancy in advanced cancer: attitudes of patients, family members, and other healthcare professionals
Source: Support Care Cancer. 2022 Jun 15;30(9):7763–72. doi: 10.1007/s00520-022-07167-3 (PMC9385826; doi:10.1007/s00520-022-07167-3)
Supplement: Supplementary file 1 — Supplementary file1 (DOCX 67 KB) [file 520_2022_7167_MOESM1_ESM.docx]

**Title: Using three scenarios to explain life expectancy in advanced cancer: attitudes of patients, family members, and other healthcare professionals**

# Journal: The Journal of Supportive Care in Cancer

**Authors:** Sharon H. Nahm^1-2^, Martin R. Stockler^1-3^, Andrew J. Martin¹, Chris Brown¹, Peter Grimison^2,4-5^, Peter Fox^6^, Rob Zielinski ^6^, Geoffrey AT. Hawson^7^, Martin HN. Tattersall*^2,4^, Belinda E. Kiely^1-3,8^

***In tribute:** Prof Martin H.N. Tattersall 1941-2020. For his pioneering role in medical oncology and research to enhance patient-doctor communication and shared decision-making.

**Affiliations**

1. The NHMRC Clinical Trials Centre, University of Sydney, Sydney, Australia; 2. Sydney Medical School, University of Sydney, Sydney, Australia; 3. Concord Cancer Centre, Sydney, Australia; 4. Chris O’Brien Lifehouse, Sydney, Australia; 5. Alan Coates Cancer Centre, Dubbo, Australia; 6. Central West Cancer Care Centre, Orange, Australia; 7. Nambour General Hospital, Nambour, Australia; 8. Macarthur Cancer Therapy Centre, Sydney, Australia

**Corresponding author:** Belinda.kiely@sydney.edu.au

# Supplementary Text 1 – Example of printed summary of survival information

**Imagine 100 people…**

It is impossible to see into the future but we can get an idea of how the future might look by thinking about how a group of similar people would do.

Many people in this situation have said it's helpful to think about the best case scenario, the worst case scenario, and the most likely scenario, which is somewhere in between.

If we imagine 100 people in exactly the same situation, then we'd expect:

  - the 5 to 10 who did best would live longer than 3 years

  - the 5 to 10 who did worst would die within 3 months

  - the middle 50 would live 6 months to 2 years.

This also means that
 - half the people would live longer than 12 months and
 - half the people would live less than 12 months.

Cancer is very unpredictable. Sometimes that unpredictability acts in your favour.

**Number of people**

**Supplementary Text 2- Oncologist questionnaire after consultation**

The following questions are designed to determine your experience using the iTool to estimate and explain survival time with *this patient*.

| **Please circle one number for each line to show how you feel about that statement** | | | | | | |
| --- | --- | --- | --- | --- | --- | --- |
| **Statement** | | **Strongly Disagree** | **Disagree** | **Unsure** | **Agree** | **Strongly Agree** |
| **1.** | **It was difficult to estimate the “median” survival to enter into the iTool for this patient** | 1 | 2 | 3 | 4 | 5 |
| **2.** | **For me, explaining life expectancy as 3 scenarios:** |  |  |  |  |  |
| a | was helpful | 1 | 2 | 3 | 4 | 5 |
| b | was difficult | 1 | 2 | 3 | 4 | 5 |
| c | was stressful | 1 | 2 | 3 | 4 | 5 |
| d | significantly lengthened the consultation | 1 | 2 | 3 | 4 | 5 |
| e | was facilitated by using the iTool | 1 | 2 | 3 | 4 | 5 |
| **3.** | **Using the iTool during the consultation was intrusive** | 1 | 2 | 3 | 4 | 5 |
| **4.** | **For the patient, having life expectancy explained this way was:** |  |  |  |  |  |
| a | helpful | 1 | 2 | 3 | 4 | 5 |
| b | reassuring | 1 | 2 | 3 | 4 | 5 |
| c | upsetting | 1 | 2 | 3 | 4 | 5 |
| d | too complicated | 1 | 2 | 3 | 4 | 5 |
| e | improved their understanding | 1 | 2 | 3 | 4 | 5 |
| **5.** | **The information was helpful for the other family member(s) or carer(s) in the room  (leave blank if no other people)** | 1 | 2 | 3 | 4 | 5 |

**Supplementary Text 3- Patient questionnaire**

1. Age in years: __ __

2. Sex: 🞏 M 🞏 F

3. Highest education level achieved: (please select one of the options below)

🞏 Year 10 or less

🞏 Completed high school

🞏 College diploma, university degree or other post high school qualification

4. Year your cancer was first diagnosed: __ __ __ __

5. What part of the body did your cancer start in (please tick only one of the boxes):

🞏 Breast

🞏 Lung

🞏 Bowel (colon or rectum)

🞏 Prostate

🞏 Ovary, uterus (womb) or cervix

🞏 Bladder

🞏 Kidney

🞏 Oesophagus

🞏 Stomach

🞏 Pancreas

🞏 Mouth, tongue or throat

🞏 Skin (melanoma)

🞏 Other

🞏 Unsure

| **Please circle one number for each line to show how you feel about that statement** | | | | | | |
| --- | --- | --- | --- | --- | --- | --- |
| **6.** | **Having my life expectancy explained this way:** | **Strongly Disagree** | **Disagree** | **Unsure** | **Agree** | **Strongly Agree** |
| a | made sense (the information was clear and easy to understand) | 1 | 2 | 3 | 4 | 5 |
| b | will help me make plans for the future | 1 | 2 | 3 | 4 | 5 |
| c | gives me hope | 1 | 2 | 3 | 4 | 5 |
| d | was reassuring | 1 | 2 | 3 | 4 | 5 |
| e | was upsetting | 1 | 2 | 3 | 4 | 5 |
| f | improved my understanding | 1 | 2 | 3 | 4 | 5 |
| g | made me feel worried or anxious | 1 | 2 | 3 | 4 | 5 |
| h | was helpful | 1 | 2 | 3 | 4 | 5 |
| **7.** | **Being told the *best case scenario* was helpful** | 1 | 2 | 3 | 4 | 5 |
| **8.** | **Being told the *worst case scenario* was helpful** | 1 | 2 | 3 | 4 | 5 |
| **9.** | **Being told the *most likely scenario* was helpful** | 1 | 2 | 3 | 4 | 5 |
| **10.** | **Being told the time *half a group of people would live longer or shorter than* was helpful** | 1 | 2 | 3 | 4 | 5 |
| **11.** | **I found this way of presenting information about life expectancy helpful** | 1 | 2 | 3 | 4 | 5 |
| **12.** | **It was helpful for me to receive a printed summary of this information** | 1 | 2 | 3 | 4 | 5 |
| **13.** | **It would be helpful for my family members to receive this information** | 1 | 2 | 3 | 4 | 5 |
| **14.** | **It would be helpful for my family doctor (GP) to receive this information** | 1 | 2 | 3 | 4 | 5 |

15. Overall, how did the information your doctor explained about your life expectancy compare with what you thought before this discussion (please tick only one box)?

Better than expected

Worse than expected

About the same as expected

16. Given the choice, which scenario would you prefer to be told about first (please tick only one box)?

Best case

Worst case

Most likely range

The order is not important to me

17. Do you plan to show (or have you already shown) the print out about your life expectancy to your family, friends or carers (please tick only one box)?

Yes

No

Please read each statement and then **circle the most appropriate number to the right** of the statement to indicate how you feel **right now**, at this moment. There is no right or wrong answer. Do not spend too much time on any of the statements but give the answer which seems to describe your present feelings best.

| Statement | | Not at all | Somewhat | Moderately | Very much |
| --- | --- | --- | --- | --- | --- |
| 18. | I feel calm | 1 | 2 | 3 | 4 |
| 19. | I am tense | 1 | 2 | 3 | 4 |
| 20. | I feel upset | 1 | 2 | 3 | 4 |
| 21. | I am relaxed | 1 | 2 | 3 | 4 |
| 22. | I feel content | 1 | 2 | 3 | 4 |
| 23. | I am worried | 1 | 2 | 3 | 4 |

Please read each statement below and then **circle the most appropriate number to the right** of the statement to indicate how much you agree with that statement. Try not to let your response to one statement influence your responses to other statements.  There are no "correct" or "incorrect" answers.  Answer according to your own feelings, rather than how you think "most people" would answer.

| **Statement** | | Strongly  Disagree | Disagree | Agree | Strongly  Agree |
| --- | --- | --- | --- | --- | --- |
| 24. | I have a positive outlook toward life | 1 | 2 | 3 | 4 |
| 25. | I have short and/or long range goals | 1 | 2 | 3 | 4 |
| 26. | I feel all alone | 1 | 2 | 3 | 4 |
| 27. | I can see possibilities in the midst of difficulties | 1 | 2 | 3 | 4 |
| 28. | I have a faith that gives me comfort | 1 | 2 | 3 | 4 |
| 29. | I feel scared about my future | 1 | 2 | 3 | 4 |
| 30. | I can recall happy/joyful times | 1 | 2 | 3 | 4 |
| 31. | I have deep inner strength | 1 | 2 | 3 | 4 |
| 32. | I am able to give and receive caring/love | 1 | 2 | 3 | 4 |
| 33. | I have a sense of direction | 1 | 2 | 3 | 4 |
| 34. | I believe that each day has potential | 1 | 2 | 3 | 4 |
| 35. | I feel my life has value and worth | 1 | 2 | 3 | 4 |

Please read each statement and then **circle the most appropriate number to the right** of the statement to indicate how much you agree with that statement.  Try not to let your response to one statement influence your responses to other statements.  There are no "correct" or "incorrect" answers.  Answer according to your own feelings, rather than how you think "most people" would answer.

| Statement | | Strongly Disagree | Disagree | Unsure | Agree | Strongly Agree |
| --- | --- | --- | --- | --- | --- | --- |
| 36. | In uncertain times, I usually expect the best | 1 | 2 | 3 | 4 | 5 |
| 37. | It's easy for me to relax | 1 | 2 | 3 | 4 | 5 |
| 38. | If something can go wrong for me, it will. | 1 | 2 | 3 | 4 | 5 |
| 39. | I'm always optimistic about my future | 1 | 2 | 3 | 4 | 5 |
| 40. | I enjoy my friends a lot | 1 | 2 | 3 | 4 | 5 |
| 41. | It's important for me to keep busy | 1 | 2 | 3 | 4 | 5 |
| 42. | I hardly ever expect things to go my way. | 1 | 2 | 3 | 4 | 5 |
| 43. | I don't get upset too easily | 1 | 2 | 3 | 4 | 5 |
| 44. | I rarely count on good things happening to me | 1 | 2 | 3 | 4 | 5 |
| 45. | Overall, I expect more good things to happen to me than bad | 1 | 2 | 3 | 4 | 5 |

46. Did you find taking part in this study distressing?

🞏 Not at all

🞏 Somewhat

🞏 Moderately

🞏 Extremely

Please let us (the researchers, your doctor or nurse) know if you would like to talk about the information with your doctor or anyone else

Thank you for helping with this study. This is the last page.

**Supplementary Text 4- Family member questionnaire**

1. Age:

2. Sex: 🞏 M 🞏 F

3. Which of the following best describes your relationship to the patient: (please tick only one of the boxes):

🞏 husband / wife / partner

🞏 son / daughter

🞏 son in law / daughter-in-law

🞏 brother / sister

🞏 mother / father

🞏 niece / nephew

🞏 care giver

🞏 other (please specify)

| **Please circle one number for each line to show how you feel about that statement** | | | | | | |
| --- | --- | --- | --- | --- | --- | --- |
| **4.** | **Having life expectancy explained as three possible scenarios:** | **Strongly Disagree** | **Disagree** | **Unsure** | **Agree** | **Strongly Agree** |
| a | was helpful | 1 | 2 | 3 | 4 | 5 |
| b | made sense (the information was clear and easy to understand) | 1 | 2 | 3 | 4 | 5 |
| c | was too complicated | 1 | 2 | 3 | 4 | 5 |
| d | helps me make plans for the future | 1 | 2 | 3 | 4 | 5 |
| e | gives me hope | 1 | 2 | 3 | 4 | 5 |
| f | was reassuring | 1 | 2 | 3 | 4 | 5 |
| g | was upsetting | 1 | 2 | 3 | 4 | 5 |
| h | improved my understanding | 1 | 2 | 3 | 4 | 5 |
| **5.** | **It was helpful to receive a printed summary of the three scenarios** | 1 | 2 | 3 | 4 | 5 |

**Supplementary Text 5 – Healthcare professional questionnaire**

| **1. Age:** | __ __ years |  |
| --- | --- | --- |
| **2. Sex:** | 🞏 Male | 🞏 Female |
| **3. Specialty type (please tick only one of the boxes below):** | | |
| 🞏 Surgical | 🞏 Radiation oncology | 🞏 Medical oncology |
| 🞏 Palliative care | 🞏 Other internal medical | 🞏 Nurse |
| 🞏 Psychologist | 🞏 Social worker | 🞏 Other |
| **4. What proportion of the patients in your care have been diagnosed with cancer?** | | |
| 🞏 less than one third | 🞏 one third to two thirds | 🞏 more than two thirds |
| **5. The *best case* scenario was:** | | |
| 🞏 better than I expected | 🞏 worse than I expected | 🞏 about the same as I expected |
| **6. The *worst case* scenario was:** | | |
| 🞏 better than I expected | 🞏 worse than I expected | 🞏 about the same as I expected |
| **7. The *most likely* scenario was:** | | |
| 🞏 better than I expected | 🞏 worse than I expected | 🞏 about the same as I expected |
| **8. The *median* survival time (time when 50% of the people have died) was:** | | |
| 🞏 better than I expected | 🞏 worse than I expected | 🞏 about the same as I expected |

| **Please circle one number for each line to show how you feel about that statement** | | | | | | |
| --- | --- | --- | --- | --- | --- | --- |
| **9.** | **Receiving the 1 page summary of life expectancy information for this patient:** | **Strongly Disagree** | **Disagree** | **Unsure** | **Agree** | **Strongly Agree** |
| a | makes sense (the information is clear and easy to understand) | 1 | 2 | 3 | 4 | 5 |
| b | will help me make management and treatment decisions | 1 | 2 | 3 | 4 | 5 |
| c | improves my understanding of their prognosis | 1 | 2 | 3 | 4 | 5 |
| d | will help me answer questions from my patient about prognosis | 1 | 2 | 3 | 4 | 5 |
| e | is more informative than the prognostic information I usually receive for my patients with advanced cancer | 1 | 2 | 3 | 4 | 5 |
| **10.** | **I found receiving this format of information on prognosis helpful** | 1 | 2 | 3 | 4 | 5 |
| **11.** | **It would be helpful for me to receive this information my other patients with advanced cancer** | 1 | 2 | 3 | 4 | 5 |
| **12.** | **I think my patients would find this information** |  |  |  |  |  |
| a | helpful | 1 | 2 | 3 | 4 | 5 |
| b | distressing | 1 | 2 | 3 | 4 | 5 |
| c | reassuring | 1 | 2 | 3 | 4 | 5 |
| **13.** | **It would be helpful for other patients with advanced cancer to receive this information** | 1 | 2 | 3 | 4 | 5 |

**Is there any other information you would like to see included?**

**Supplementary Text 6- Oncologist questionnaire post study**

The following questions are designed to determine your attitudes to using the iTool throughout the study when discussing prognosis with your patients.

| **Please circle one number for each line to show how you feel about that statement** | | | | | | |
| --- | --- | --- | --- | --- | --- | --- |
| **Statement** | | **Strongly Disagree** | **Disagree** | **Unsure** | **Agree** | **Strongly Agree** |
| **1.** | **I found using the iTool:** |  |  |  |  |  |
| a | helpful | 1 | 2 | 3 | 4 | 5 |
| b | easy | 1 | 2 | 3 | 4 | 5 |
| c | made discussing prognosis easier | 1 | 2 | 3 | 4 | 5 |
| d | made me more prepared to discuss prognosis | 1 | 2 | 3 | 4 | 5 |
| e | makes me more likely to discuss prognosis | 1 | 2 | 3 | 4 | 5 |
| f | improved the way I explain prognosis | 1 | 2 | 3 | 4 | 5 |
| g | improved the way I document prognosis in my notes and letters | 1 | 2 | 3 | 4 | 5 |
| **2.** | **I would like to continue using the iTool** | 1 | 2 | 3 | 4 | 5 |
